# Controlling the Adsorption of β-Glucosidase onto Wrinkled SiO_2_ Nanoparticles To Boost the Yield of Immobilization of an Efficient Biocatalyst

**DOI:** 10.1021/acs.langmuir.2c02861

**Published:** 2023-01-18

**Authors:** Giulio Pota, Noemi Gallucci, Domenico Cavasso, Irene Russo Krauss, Giuseppe Vitiello, Fernando López-Gallego, Aniello Costantini, Luigi Paduano, Valeria Califano

**Affiliations:** † University of Naples Federico II, Department of Chemical, Materials and Production Engineering, 80125Naples, Italy; ‡ University of Naples Federico II, Department of Chemical Sciences, 80125Naples, Italy; § CSGI, Center for Colloid and Surface Science, 50019Sesto Fiorentino(FI), Italy; ∥ Center for Cooperative Research in Biomaterials (CIC BiomaGUNE), Basque Research and Technology Alliance (BRTA), 20850Donostia-San Sebastián, Spain; ⊥ Ikerbasque, Basque Foundation for Science, 948009Bilbao, Spain; # Institute of Sciences and Technologies for Sustainable Energy and Mobility (STEMS), National Research Council of Italy (CNR), Viale Marconi 4, 80125Naples, Italy

## Abstract

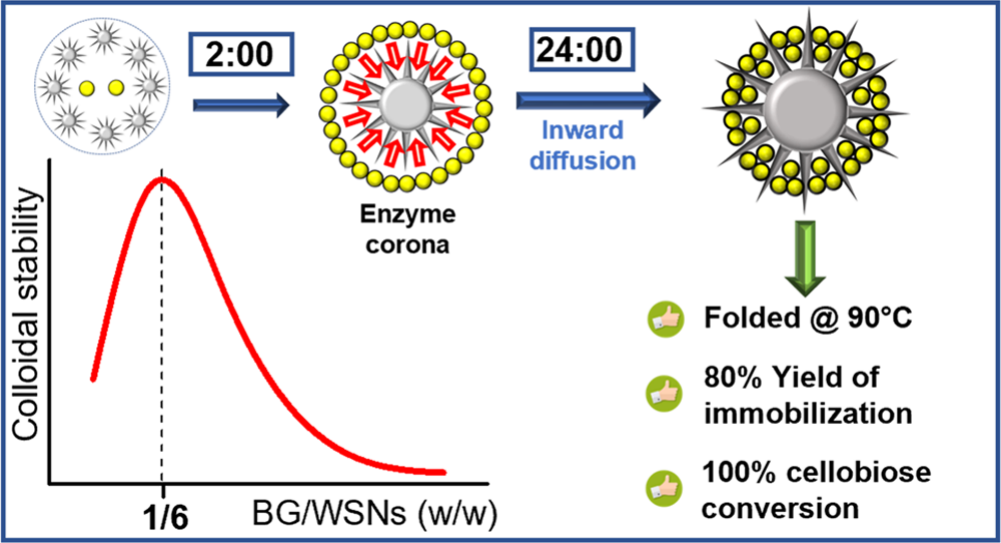

β-Glucosidase (BG) catalyzes the hydrolysis of cellobiose to glucose, a substrate for fermentation to produce the carbon-neutral fuel bioethanol. Enzyme thermal stability and reusability can be improved through immobilization onto insoluble supports. Moreover, nanoscaled matrixes allow for preserving high reaction rates. In this work, BG was physically immobilized onto wrinkled SiO_2_ nanoparticles (WSNs). The adsorption procedure was tuned by varying the BG:WSNs weight ratio to achieve the maximum controllability and maximize the yield of immobilization, while different times of immobilization were monitored. Results show that a BG:WSNs ratio equal to 1:6 wt/wt provides for the highest colloidal stability, whereas an immobilization time of 24 h results in the highest enzyme loading (135 mg/g of support) corresponding to 80% yield of immobilization. An enzyme corona is formed in 2 h, which gradually disappears as the protein diffuses within the pores. The adsorption into the silica structure causes little change in the protein secondary structure. Furthermore, supported enzyme exhibits a remarkable gain in thermal stability, retaining complete folding up to 90 °C. Catalytic tests assessed that immobilized BG achieves 100% cellobiose conversion. The improved adsorption protocol provides simultaneously high glucose production, enhanced yield of immobilization, and good reusability, resulting in considerable reduction of enzyme waste in the immobilization stage.

## 1 Introduction

Enzymes are a family of nontoxic, environmentally friendly biomolecules, involved in a plethora of biochemical processes.^1^ They are widely used as biocatalysts owing to their outstanding properties such as being effective under milder reaction conditions, higher specificity and selectivity, and faster kinetics with respect to traditional catalysts.^1^ However, they suffer from intrinsic instability under harsh operative conditions and are expensive.^2^ Several technical challenges need to be overcome to make enzymatic processes economically feasible: the high cost of the enzymes, their low thermal and pH stability causing a loss of activity during the process, the inhibition by reactants and products, and difficult recovery.^2^ These drawbacks can be overcome by enzyme immobilization. Indeed, immobilization usually results in increased pH, temperature, and organic solvent tolerance as well as resistance to proteolytic digestion and denaturants.^3,4^ The key issue for enzyme immobilization is the selection of the immobilization technique and of the appropriate support. Many different immobilization methods are proposed to improve the biocatalyst efficiency.^5^ Among them, physical immobilization is the simplest and can be carried out under mild conditions,^6^ being based on physical interactions, such as hydrogen bonding, electrostatic forces, and hydrophobic interactions between the enzyme and the matrix. With this method, the enzyme activity is often preserved, but the immobilized enzyme can have poor operation stability and be subjected to leaching.^7^ For this reason, the choice of a good support is crucial. It should exhibit thermal and mechanical stability, high surface area, adequate pore diameter, biocompatibility, and chemical affinity toward the enzyme, to create the optimal microenvironment to preserve protein conformation and activity and ensure reusability.^6^ In this context, mesoporous SiO_2_ nanoparticles are very good supports, owing to a high surface area and tunable porosity allowing for the high loading of guest species.^8−10^ Moreover, the great availability of surface hydroxyl groups enables easy chemical functionalization.^11−14^ In particular, wrinkled silica nanoparticles (WSNs), which are mesoporous nanoparticles with central-radial pore structure, are gaining great attention as carriers for enzymes because the conical pore shape helps reduce pore blocking.^15^ Furthermore, hierarchical trimodal porosity effectively lowers diffusive limitations for both substrate and products.^15^ Another important issue is about the colloidal stability of the supported systems that has a significant effect on the catalytic performances of the immobilized enzymes.^16,17^ Indeed, fast self-aggregation or precipitation processes in the reaction media can hinder the substrate access or induce unfavorable conformational transition of the enzyme on the support,^16,18^ thus drastically decreasing the biocatalytic activity. These dynamics are often triggered by the complex behavior of enzymes in solution because proteins can unfold and aggregate, depending on ionic force and pH values, forming clusters of different sizes.^19^ Hence, robust immobilization on the nanoparticles as well as great colloidal and structural stability appears mandatory to design biocatalysts with high performances, reduce preparation costs, and promote higher reusability.^20,21^

Protein–nanoparticle interactions have been extensively studied.^22−24^ Most nanoparticles are readily covered by a dynamic layer of proteins when put in contact, generating what is called a protein corona. No single kind of interaction can be attributed to the protein–surface adsorption but rather it is generated from a complex interplay of polar and nonpolar interaction mechanisms.^22^ Both kinds of interaction can be attractive or repulsive, determining the formation of the corona. With porous nanoparticles, the protein corona that possibly forms can later migrate inside the pores.^25^

Recently, we have used WSNs as a matrix to immobilize β-glucosidase (BG) and cellulase.^26,27^ BG belongs to the glycosyl hydrolase family that finds applications in many biotechnological fields.^28,29^ It plays a key role in the enzymatic degradation of cellulose, hydrolyzing cellobiose to two glucose molecules and allowing the production of sugars that can be fermented to ethanol. The alcohol thus produced can be used as biofuel, with both environmental and geopolitical benefits.^30^ Physical immobilization was carried out to attach BG onto WSNs, leading to a performing and stable biocatalyst for the hydrolysis of cellobiose.^26^ Adsorption allowed for preserving the enzyme native conformation and increasing substrate–enzyme affinity, leading to 100% cellobiose conversion in 2 h.^31^ The yield of immobilization (YI), defined as the percentage weight ratio between the adsorbed enzyme and the overall enzyme used in the immobilization step, reached 30%.^26^ In a subsequent work dealing with the immobilization of cellulase onto the same nanoparticles, Costantini et al. found out that the YI varies with the enzyme concentration in the adsorption environment following an exponential decay function.^27^ This result confirmed what was previously observed for lysozyme immobilization into mesoporous silica.^32^ Therefore, the lower the enzyme concentration, the higher the YI and thus the lower the enzyme waste. In this work, physical immobilization of BG onto WSNs under diluted conditions was performed. Different enzyme concentrations, corresponding to precise BG:WSNs weight ratios, were investigated with the aim to discover the best conditions to limit the self-aggregation process and enhance the control over the protein–support interaction dynamics. At the same time, the search for the optimal system was intended to optimize the yield of immobilization to keep a high enzyme density over the entire surface of the nanoparticles. The most stable BG/WSNs systems were tested in the hydrolysis of cellobiose to glucose and compared with the performances of the reference system previously designed.

## 2 Experimental Section

### 2.1 Materials

Tetraethyl orthosilicate (TEOS), urea, cetyltrimethylammonium bromide (CTAB), cyclohexane, anhydrous 2-propanol (ACS reagent), ethanol, hydrochloric acid solution (37.0% wt. in water), β-glucosidase from almonds (molecular weight 135 kDa for the dimer, product number 49290, specific activity ≥4 U/mg, measured as micromoles of glucose liberated per minute at pH 5 and 37 °C with salicin as substrate), citric acid, trisodium citrate dihydrate, sulfuric acid (95.0–98.0% wt.), glucose oxidase–peroxidase (GOD-POD) assay kit, and potassium bromide were purchased from Sigma-Aldrich (Milan, Italy).

### 2.2 Synthesis of Wrinkled SiO_2_ Nanoparticles (WSNs)

The preparation of wrinkled SiO_2_ nanoparticles (WSNs) was inspired by the synthetic route described by Moon and Lee,^15^ which was opportunely modified by using cetyltrimethylammonium bromide (CTAB) instead of cetylpyridinium bromide (CPB) as templating agent for mesopore formation.^33^ Also, a more accurate 24-h lasting surfactant removal step was introduced into the preparation protocol. More specifically, 123.68 mL of a solution of IPA and cyclohexane (IPA 3 v/v%) was mixed into an aqueous solution of CTAB (0.01 M) and urea (0.33 M). The reaction mixture promptly turned from transparent into white. Afterward, TEOS was added dropwise to the stirred solution for a final concentration of 0.18 M. Finally, the reaction system was stirred for 30 min at room temperature and then heated to 70 °C for 16 h. The obtained nanoparticles were centrifuged, washed three times with ethanol, and subjected to acid extraction of the surfactant by dispersion in a HCl–ethanol solution ([HCl] = 1.3 M) for 24 h at 70 °C. Finally, the nanoparticles were collected by centrifugation and washed three times with ethanol.

### 2.3 Physical Immobilization of BG onto WSNs

Physical immobilization of BG onto WSNs was designed following the protocol reported by Califano et al.^26^ However, to define the optimal conditions for enzyme adsorption preventing the self-aggregation process, the procedure was carried out in diluted conditions and different concentrations of BG were investigated. More precisely, 3 mg of WSNs was dispersed in 9.5 mL of citric acid/sodium citrate buffer (21 mM, pH = 5). A 500 μL amount of each BG solution in buffer was then added to the WSN colloidal suspension. Four BG solutions of different concentrations were tested: 0.6, 1, 1.5, and 3 mg/mL, corresponding to precise BG:WSNs weight ratios of 1:10, 1:6, 1:4, and 1:2, respectively. Each mixture was kept under mild stirring (400 rpm) at 40 °C for 24 h. Then 0.6 mL of each prepared BG/WSN mixture was analyzed through dynamic light scattering (DLS) to identify the best immobilization conditions for enhancing the stability of the supported enzyme. Subsequently, to study the time evolution of the most controllable BG/WSNs system (1:6), 0.6 mL of each prepared BG/WSN mixture was withdrawn after 15 min, 2 h, 6 h, and 24 h to be analyzed through DLS, circular dichroism (CD), and ζ-potential measurements. The prepared samples were named as BG/WSNs_15 min, BG/WSNs_2h, BG/WSNs_6h, and BG/WSNs_24h. The supported BG/WSNs biocatalysts were collected by centrifugation after double-washing with bidistilled water to perform catalytic assays as well as other physicochemical analyses. This optimized BG/WSNs system was studied in terms of catalytic performances. The yield of immobilization (YI) was evaluated through thermogravimetric analysis (TGA).

### 2.4 Physicochemical Analysis of Morphology, Size Distribution and Solution Behavior of BG/WSNs

Morphological and dimensional analysis of bare WSNs and BG-loaded WSNs was carried out through transmission electron microscopy (TEM), using a FEI Tecnai G12 Spirit Twin (FEI, Hillsboro, OR) with a LaB6 emission source and an acceleration tension of 120 kV. The images are taken with a CCD FEI Eagle 4k camera. The samples to be measured were prepared by soaking the proper copper grid used for TEM measurements (400 mesh with a thin carbon film) in an aqueous suspension of the nanoparticles with the concentration set at 0.5 mg/mL. Time evolution of the colloidal stability and self-aggregation process of the BG/WSNs systems during the immobilization process was monitored by DLS measurements.^34,35^ A homemade experimental set up, composed of a Photocor compact goniometer (Moscow, Russia), a SMD 6000 laser Quantum 50 mW light source (Laser Quantum, Fremont, CA) operating at 532.5 Å, a photomultiplier (PMT-120-OP/B), and a correlator (Flex02–01D) from Correlator.com (Shenzhen, China) was used. The experimental temperature was fixed to the room value (25 °C), while the scattering angle θ was set at 130°. A regularization algorithm^36^ was used to analyze the correlation function of the scattered intensity (*I*(*t*)) reported below as
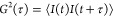
where *G*^2^(τ) is the correlation function of the scattered intensity *I*(*t*) and the angular brackets denote an average over time *t*. The autocorrelation function is necessary to extract information about the colloidal stability of the nanostructures from the random fluctuation of the scattered intensity. The hydrodynamic radius (*R*_H_) of the nanostructures was calculated as follows:
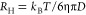
where *k*_B_ is the Boltzmann constant, *T* is the absolute temperature, η is the solution viscosity, and *D* is the average diffusion coefficient measured in the DLS experiments. For each sample, 12 acquisitions of the scattering intensities lasting 120 s each were collected to have a good and reproducible statistics.

ζ-Potential measurements were performed to assess the nature of the enzyme–support interaction and the influence of the surface charge on the colloidal stability of the BG/WSNs nanosystems. About 600 μL of each suspension at different immobilization times was analyzed by means of electrophoretic light scattering using a Zetasizer Nano ZSP (Malvern Instruments, England). Each measurement was recorded at 25 °C upon a 30 s equilibration time, and the average of three measurements at a stationary level was taken. The ζ-potential was calculated by the Smoluchowski model.

### 2.5 Quantification of the Enzyme Fraction in Commercial BG

An estimation of the protein content in the commercial BG was realized through UV analysis, following the procedure first reported by Goldfarb in the 1950s.^37^ Briefly, a 1 mg/mL BG buffer solution was loaded in a 1 cm path length quartz cuvette and subjected to UV–vis spectrocopy, recording in the 240–320 nm range. The enzyme concentration was then calculated following eq 1 derived from the Lambert–Beer law:
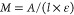
1where *M* (mol·L^–1^) is the protein molar concentration, *A* is the absorbance at 280 nm, *l* (cm) is the optical length, and ε is the BG molar absorptivity (L·mol^–1^·cm^–1^). The presence of other protein fractions within the commercial powder was investigated through sodium dodecyl sulfate–polyacrylamide gel electrophoresis (SDS-PAGE).

### 2.6 Evaluation of the Yield of Immobilization

The yield of immobilization (YI) was determined through thermogravimetric analysis (TGA). Ten milligrams of each dried sample was ground and loaded into platinum pans to be thermally treated from 30 °C to 1000 °C under air atmosphere, with a heating rate of 10 °C/min. The decay in the initial weight of each sample was monitored. The enzyme weight fraction contained in the BG/WSNSs samples was calculated as the weight loss between 200 °C and the final temperature over the initial weight, in percentage, minus the organic weight fraction of the bare support. YI was then evaluated as the percentage ratio between the loaded enzyme and the amount of protein dissolved initially in the adsorption mixture. The activity yield of immobilization YI_E_ was calculated by the formula YI_E_ = (*E*_i_/*E*_c_) × 100, where *E*_c_ represents the contacted enzyme activity and *E*_i_ the activity expressed by the immobilized enzyme.^38^

### 2.7 Conformational Analysis of Immobilized BG

Circular dichroism (CD) was carried out to analyze the structural stability of the supported BG enzyme as well as the evolution of its conformation. For CD analysis, 300 μL of each BG/WSNs suspension was withdrawn from the reactor, poured into a 0.1 cm path length cuvette, and analyzed using a Jasco J-710 spectropolarimeter equipped with a Peltier thermostatic cell holder (model PTC-348WI). CD spectra were recorded in 195–250 nm range, with a resolution of 0.5 nm, at both room temperature (25 °C) and reaction temperature (50 °C). Thermal denaturation curves were obtained by heating the samples from 25 °C to 90 °C, with a heating rate of 1 °C/min and following the CD signal at the fixed wavelength of 222 nm. A Nexus spectrometer equipped with a DTGS (deuterated triglycine sulfate) KBr detector was used to perform FTIR experiments. All the BG/WSNs samples were dried, ground, and pressed into pellets (13 nm in diameter). FTIR spectra were recorded in the 4000–400 cm^–1^ range, choosing a spectral resolution of 2 cm^–1^ and 32 scans for each acquisition. The KBr spectrum was chosen as the background. The occurrence of any modifications in the protein secondary structure was assessed by Gaussian deconvolution of amide I band, performed by means of GRAMS 32 software. The number of Gaussian components and their initial position were determined by the second derivative spectrum.

### 2.8 Catalytic Assays

For the hydrolysis of cellobiose to glucose, a cellobiose solution in citric acid/sodium citrate buffer (pH = 5, 21 mM) was added to an equal volume of a BG/WSNs suspension in the same medium to have final concentrations of cellobiose and BG fixed to 1.5 and 0.15 mg/mL, respectively. The system was kept under mild stirring at 50 °C for 24 h. The supernatant with the final obtained product was separated from the supported BG/WSNs biocatalyst by centrifugation (11 500 rpm, 10 min) and then was kept in an oven (100 °C, 10 min) to thermally inactivate traces of the free enzyme which might have leaked from the support. Finally, the concentration of produced glucose was assessed through the d-glucose oxidase–peroxidase method.^39^ In detail, 300 μL of the collected supernatant was diluted to 1:10 v/v with bidistilled water, mixed into 600 μL of glucose-measuring reagent, and kept in a thermostatically controlled water bath at 37 °C. After 30 min, the reaction was stopped by adding 600 μL of sulfuric acid (12 N), and 1.5 mL of the final solution was poured into a 1 cm path length quartz cuvette and subjected to absorbance measurement at 540 nm using a Shimadzu UV-2600i spectrophotometer (Shimadzu, Milan, Italy). The glucose concentration was estimated on the basis of a calibration curve. The results were expressed in terms of yield of cellobiose conversion, defined as the concentration (mg/mL) ratio between obtained glucose and initially loaded cellobiose, in percentage. Similarly, the product obtained after 10 min of reaction was also analyzed to determine the specific activity of the supported biocatalysts, expressed in U/mg of enzyme. Units (U) indicate the micromoles of glucose produced per minute by a certain amount of enzyme. Experiments were repeated in triplicate.

### 2.9 Operational and Thermal Stability

Reusability assays were carried out for BG/WSNs_2h and BG/WSNs_24h systems. The biocatalysts were tested in consecutive reaction cycles of 24 h. After each cycle, the produced glucose was evaluated as previously described. The biocatalysts were collected by centrifugation and washed twice with bidistilled water before each reaction cycle. The results were expressed in terms of glucose production over the reuse cycles. The occurrence of leakage phenomena affecting the performance of the supported biocatalysts in the consecutive reuses was assessed by TGA measurements. The experimental conditions were the same as those used to evaluate the yield of immobilization. More specifically, the enzyme weight fraction was estimated before and after the reuse cycle associated with a remarkable loss in terms of glucose production.

Both the supported biocatalysts and free BG underwent thermal stability assessment. Briefly, the samples were dispersed (dissolved, in the case of the free enzyme) in citrate buffer, incubated for 1 h at a set temperature (60 °C, 70 °C, or 80 °C), and then used to perform cellobiose hydrolysis for 24 h at 50 °C. The cellobiose conversion obtained without subjecting the samples to thermal stress was chosen as the reference to evaluate the residual cellobiose conversion (%).

## 3 Results and Discussion

### 3.1 Colloidal Behavior and Morphology of Immobilized BG/WSN

**Figure 1:**
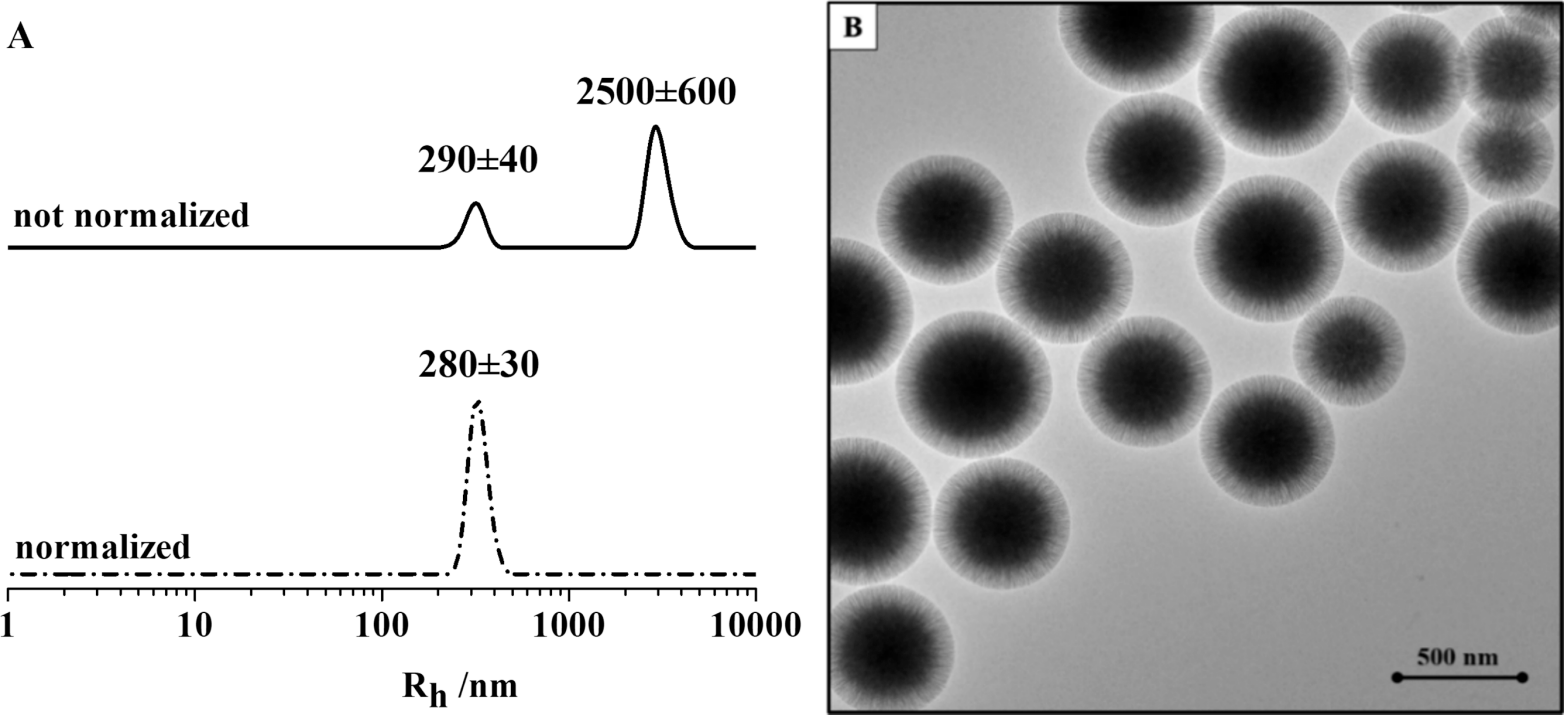
A. Hydrodynamic radius distribution of bare WSNs: intensity-weighted profile (solid line) and numerical-weighted profile (dashed line). B. TEM image of bare WSNs, scalebar = 500 nm.

DLS analysis was performed on both naked and BG-loaded WSNs to investigate the colloidal behavior of the systems in an aqueous environment as a function of the enzyme/nanoparticles ratios and immobilization times. First, a suspension of bare WSNs was analyzed as reference sample. As reported in Figure 1A, the hydrodynamic radius distribution shows a polydisperse system with the presence of two populations: the first one is centered at about 290 nm, while the second one is centered at about 2500 nm. This representation emphasizes the presence of large aggregates. Converting the intensity-weighted profile into a numerical-weighted profile, an indication of the relative concentration of the different species in the WSNs suspension is given. This second representation clearly indicates the presence of the most abundant population centered at about 280 nm as the hydrodynamic radius.

Figure 1B reports TEM micrographs for bare WSNs. The nanoparticles exhibit spherical profiles, with silica fibers spreading radially from the center to the outer surface. The mesoporous structure is made of conical pore channels, with pore size increasing moving outward, as confirmed by the remarkable decrease in contrast with respect to the inner portion of the nanoparticles, where the silica skeleton gets thicker. Moreover, this micrograph confirms the presence of silica nanoparticles with sizes ranging from 450 to 550 nm in diameter, whereas micrometric aggregates are not detected. Therefore, the population of 2500 nm in diameter detected through DLS analysis can be univocally attributed to the presence of clusters of WSNs, confirming that the naked nanostructures tend to aggregate in aqueous solution. As described in Experimental Section, different BG:WSNs weight ratios, equal to 1:2, 1:4, 1:6, and 1:10, were considered. In all cases, the immobilization time of 24 h was first considered, according to the previously investigated system.^26^ The total protein content in the BG commercial powder had been evaluated before the adsorption protocol was started, and the estimated value was equal to 24 wt % (see Supporting Information, Figure S1). This estimation was considered as correct because SDS PAGE analysis was performed for the 1:6 BG:WSNs ratio (Figure S2). Indeed, the images of the gels proved the absence of other proteins besides BG in the commercial product. As a matter of fact, the profiles of both the offered and the immobilized protein (Figure S2a, S2c) exhibit only one band centered at a molecular weight of about 65 kDa, corresponding to the monomeric form of BG. In fact, SDS PAGE, as known, does not allow detecting oligomeric forms of proteins due to the strong denaturating effect of SDS.^40^ No band is detected in the profile of the supernatant (Figure S2b), suggesting almost complete immobilization of the protein.

Therefore, only one-quarter of the commercial product is actually made of protein. Figure S3 displays the autocorrelation functions versus the time of BG/WSNs_24h at the considered weight ratios. However, although the self-aggregation and precipitation of greater aggregates occur in all samples, some differences can be observed as a function of the enzyme/nanoparticles weight ratio. Indeed, by comparing the autocorrelation functions shown in Figure S3, a slightly better situation is observed for 1:4 and 1:6 ratios for which the curves tend to reach a plateau condition over time, suggesting that they represent the best conditions capable of promoting greater control of the physical immobilization process of the enzyme onto WSNs. On the other hand, the correlation function of the 1:10 w/w sample starts to decay at slightly longer τ than the two other systems and does not reach a plateau at value *g*^2^(*t*) = 1, indicating the presence of greater particles, such as large clusters. This could be related to the presence of a very small fraction of WSNs covered with the BG enzyme and, therefore, the prevalence of naked WSNs, which show a greater tendency to self-aggregate and precipitate. Consequently, according to DLS evidence and considering the opportunity to use a BG amount as low as possible to make the final biocatalyst, only the system designed by fixing BG:WSN wt/wt equal to 1:6 was further investigated.

**Figure 2:**
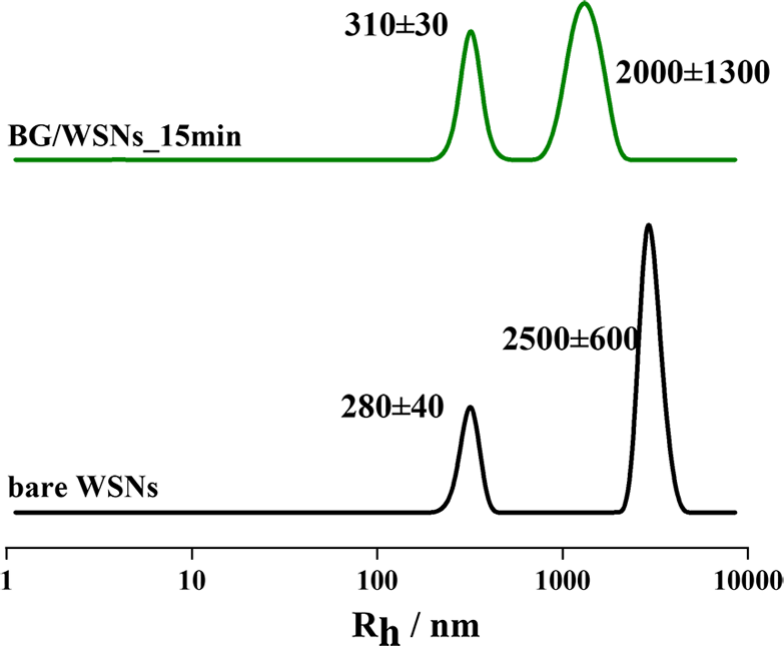
Hydrodynamic radius distribution of bare WSNs (black line) and BG/WSNs_15 min weight ratio (olive line).

Four immobilization times (15 min, 2 h, 6 h, and 24 h) were monitored by DLS to study the time evolution of the system during the adsorption process. Considering only the intensity-weighted profiles for both WSNs and BG/WSNs samples after 15 min of immobilization (Figure 2), the curves exhibit a population bigger than 2000 nm, but the most significant result is the presence of another population, centered below 500 nm, which is bigger than the corresponding one for bare WSNs. This would suggest that BG is already adsorbed onto WSNs after the first 15 min without gaining colloidal stability.

**Figure 3:**
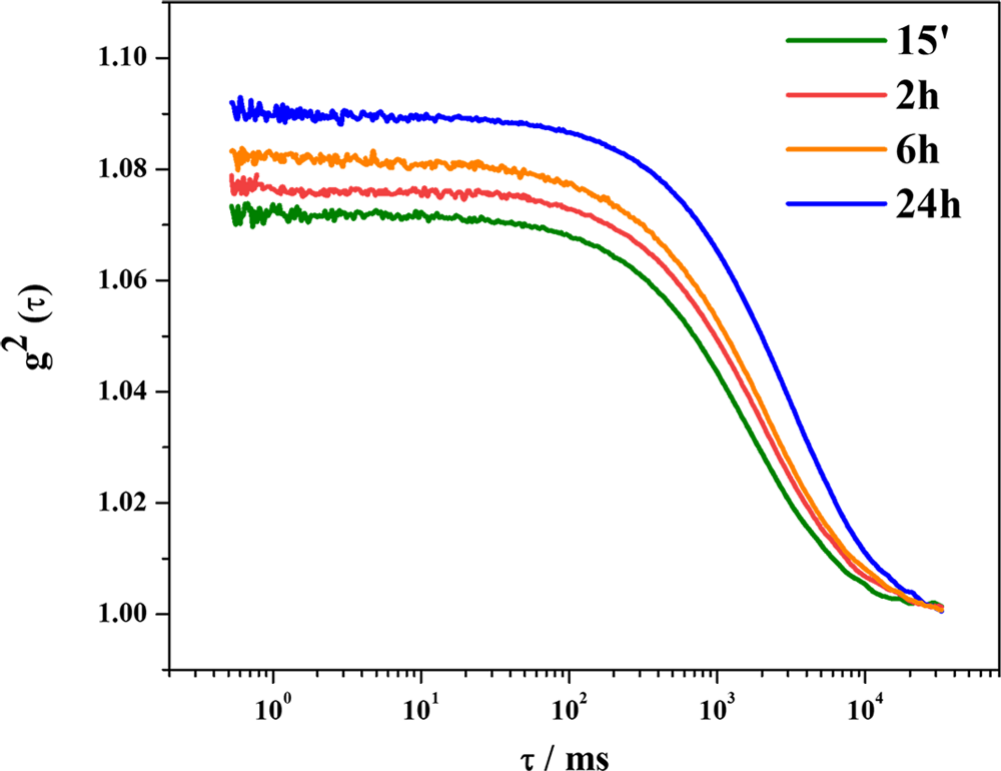
Intensity autocorrelation functions of a BG:WSNs weight ratio of 1:6 at different immobilization times: 15 min (olive line), 2 h (red line), 6 h (orange line), and 24 h (blue line).

Unfortunately, due to the rapid evolution of the system also related to the self-aggregation process occurring with the time, it is not possible to make a precise estimation of the size of BG/WSNs at diverse immobilization times. However, a comparison of the correlation functions can be done. As shown in Figure 3, no significant differences are observed between the different systems: a slightly better condition should be associated with the BG/WSNs_2h sample, which appears more similar to the BG/WSNs_15 min one, while those prepared at longer immobilization times look almost equivalent. Finally, the colloidal stability of the system could be increasingly worse with time due to aggregation phenomena triggered by adsorbed enzyme.

**Figure 4:**
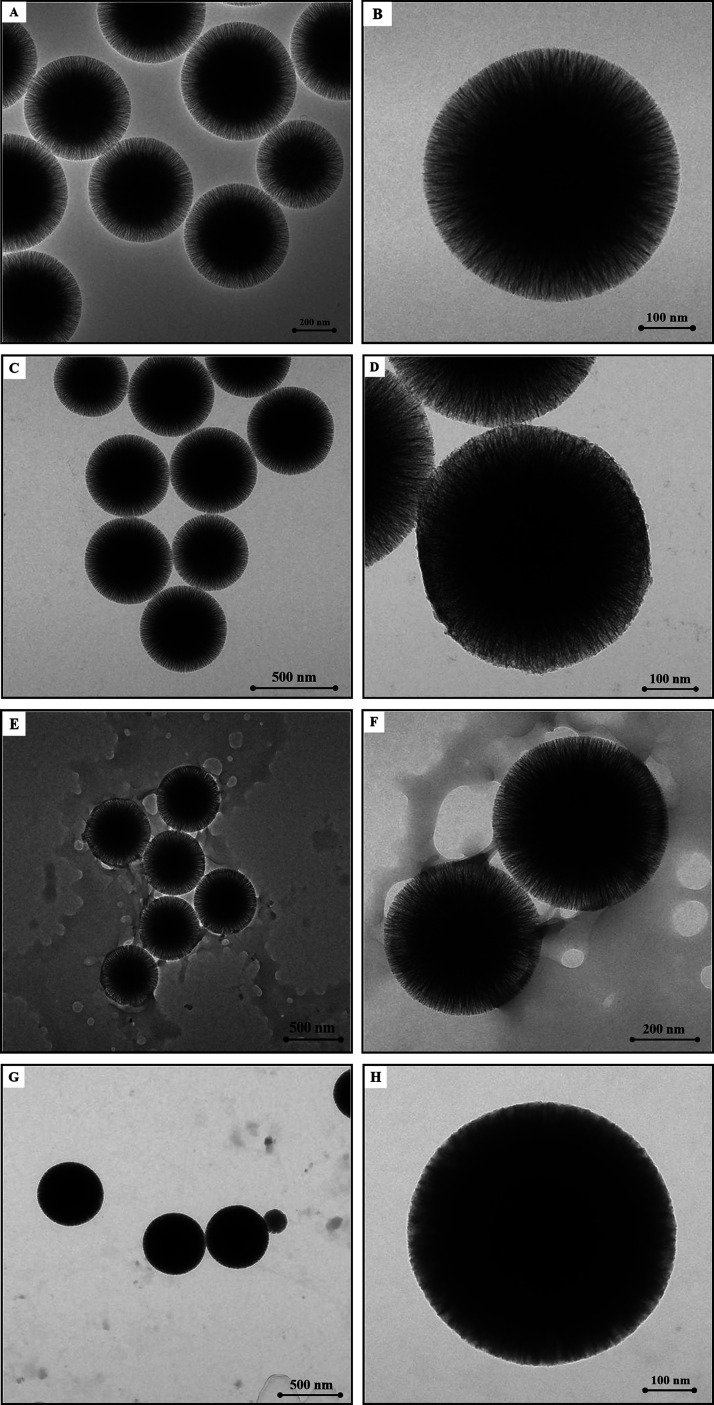
TEM images for WSNs (A scalebar = 200 nm, B scalebar = 100 nm), BG/WSNs_15 min (C scalebar = 500 nm, D scalebar = 100 nm), BG/WSNs_2h (E scalebar = 500 nm, F scalebar = 200 nm), BG/WSNs_24h (G scalebar = 500 nm, H scalebar = 100 nm).

The changes in the morphology of supported biocatalysts occurring during adsorption were investigated through TEM analysis (Figure 4). Figure 4A and 4B shows lower and higher magnifications of bare WSNs, respectively. As previously said, the pronounced difference in terms of the contrast between the core and the border portion of the nanostructure is due to the extended presence of radial pore channels. Micrographs for BG/WSN_15 min (Figure 4C, 4D) exhibit a decrease in the contrast difference. In particular, a thin enzyme layer seems to be adsorbed onto the outer surface of the nanoparticles while pores are expected to be only partially filled (Figure 4D). Moving onward to 2 h of immobilization, a wide enzyme corona surrounding clusters of nanoparticles becomes visible (Figure 4E, 4F). Indeed, 2 h are enough to allow for a consistent amount of protein to be adsorbed externally and start diffusing inward. Protein adsorption could trigger aggregation phenomena, because the enzyme appears organized in extended aggregates enveloping clusters of a few nanoparticles (Figure 4E). Furthermore, the surfaces of close nanoparticles are bound to each other by enzyme bridges (Figure 4F). Complete pore filling seems to be accomplished after 24 h. Indeed, the whole profile of the nanoparticles exhibits a homogeneously dark contrast, suggesting that the protein is completely hosted by the mesopore channels (Figure 4G). Moreover, the wide enzyme aggregates, visible in BG/WSNs_2h samples (Figure 4E, 4F), disappears, resulting in the absence of a proper protein corona layer of noticeable thickness (Figure 4H).

**Figure 5:**
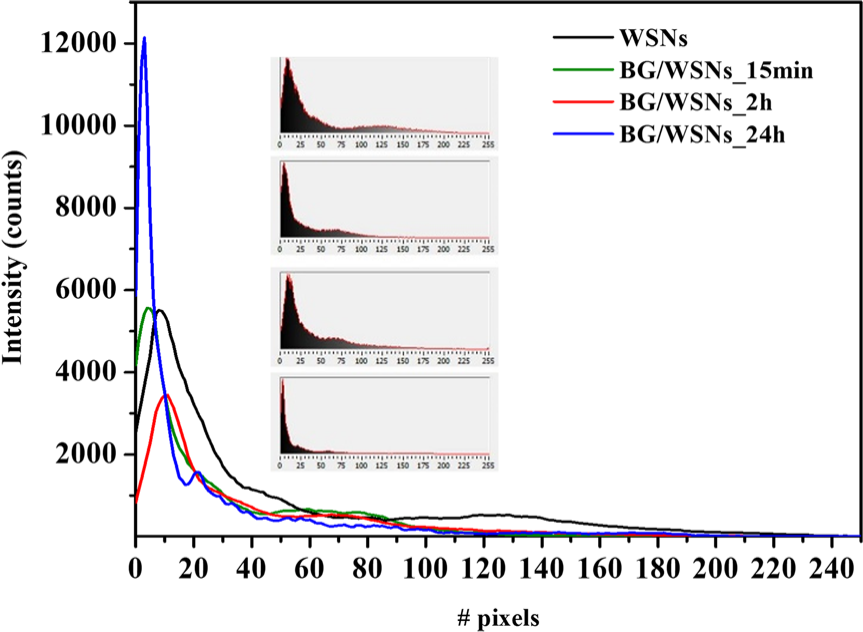
Histogram of grayscale values for WSNs, BG/WSNs_15 min, BG/WSNs_2h, and BG/WSNs_24h taken along a horizontal line passing through the center of the particle in the TEM images. Inset: normalized histograms with grayscale illustration.

A quantitative analysis of TEM images was performed by the Histogram function of the software National Instrument Vision assistant. The Histogram function counts the total number of pixels in each of the 256 grayscale levels (zero is black). These intensity profiles were taken along a horizontal line passing through the center of the particle. The results are shown in Figure 5. As can be seen, the first maximum, which represents the darkest region of the particle, moves toward smaller pixel values and increases in intensity as the contact time between the enzyme and the support increases. The second maximum, which represents the clearest part, moves significantly toward smaller pixel values (maximum at 120 for WSNs, at 70 for BG/WSNs_15 min and BG/WSNs_2h) and almost disappears for BG/WSNs_24h, meaning the entire porous structure of the silica skeleton is gradually filled by the protein during the immobilization process.

**Figure 6:**
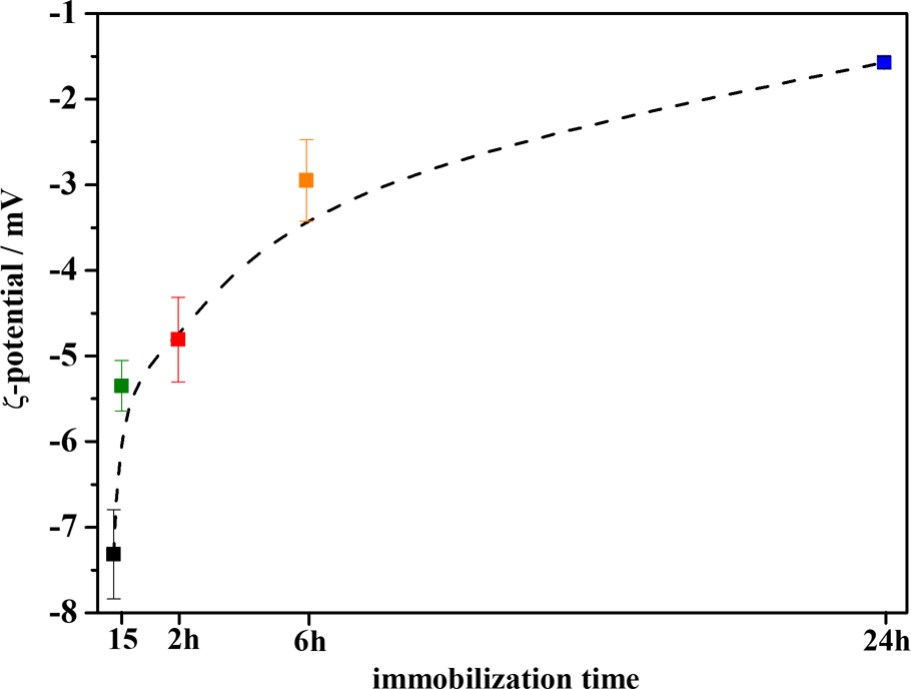
ζ-Potential values for BG/WSNs recorded during the adsorption kinetics.

ζ-Potential measurements assessed that the increasing colloidal instability of BG/WSNs nanosystems over time was due to consistent changes in the surface charge of WSNs and allowed for unveiling the mechanism of interaction between enzyme and support. Figure 6 shows the evolution of the ζ-potential during the immobilization stage. Bare WSNs exhibit a ζ-potential value equal to −7.31 mV. This is an expected result because the isoelectric point (pI) for sol–gel silica is set within a 2–3 pH interval^41−45^ below pH = 5 of citrate buffer used for the immobilization. As the adsorption process goes on, the ζ-potential rises with time from −5.35, recorded at 15 min, up to −1.57 mV, registered after 24 h. This visible trend might be evidence of the protein binding onto the silica surface because commercial BG is positively charged at pH = 5 (pI = 7.3,^46^). Previous works had relied on changes in ζ-potential values to monitor protein adsorption kinetics at the interface.^47−49^ Therefore, the YI for BG is expected to follow the same trend as the ζ-potential that is the higher the amount of adsorbed protein, the higher the increase in surface potential. In our first work dealing with the physical immobilization of BG onto WSNs, we detected the presence of hydrogen bonding between the enzyme and the silica surface.^26^ Results herein described underline that also electrostatic forces give a strong contribution to the protein–silica interaction, because the surface charge seems to be intimately correlated to the enzyme loading.

Moreover, time-dependent aggregation and thus precipitation phenomena detected through DLS analysis can be explained because the system becomes increasingly closer to the condition of zero net charge, for which no electrostatic repulsion helps to keep the individual nanostructures separate. This condition does not allow the individual nanostructures to remain separate.

### 3.2 TGA Analysis for the Estimation of the Yield of Immobilization

**Figure 7:**
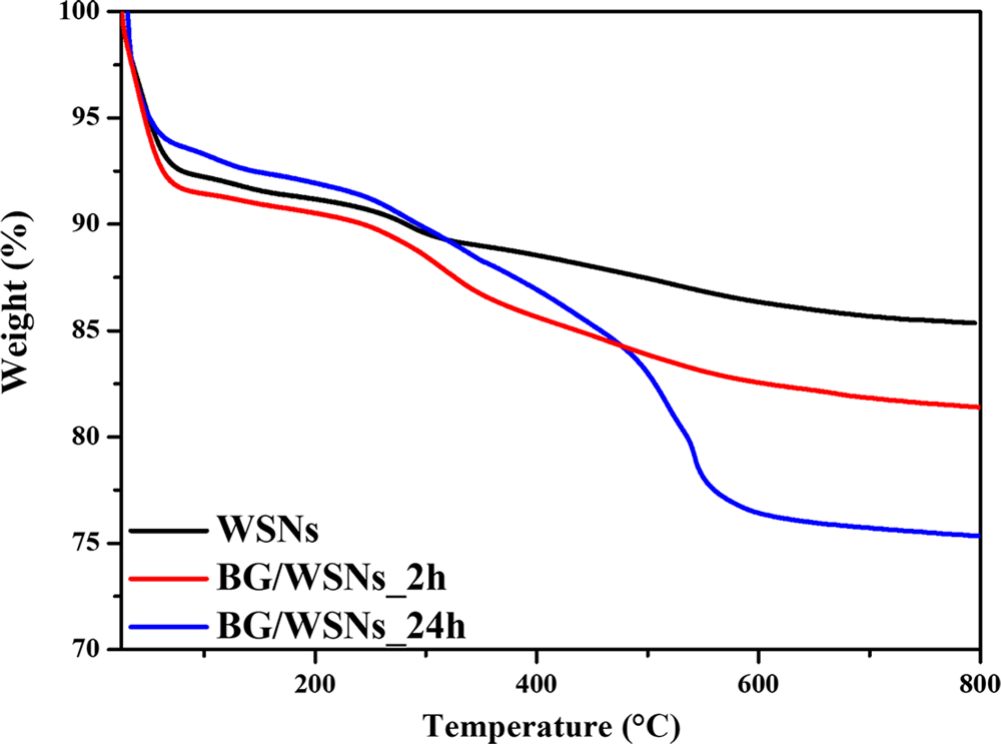
Weight loss profiles for WSNs (black curve), BG/WSNs_2h (red curve), and BG/WSNs_24h (blue curve).

YI of BG/WSNs was estimated through TGA measurements carried out after 2 and 24 h of immobilization. The reason for choosing these systems is that they were the only ones to load consistent amounts of enzyme, as revealed by the TEM images. Figure 7 reports thermograms for bare WSNs as well as BG immobilized in 2 and 24 h. WSNs experience a weight loss of 6.8% in the 200–800 °C temperature range, while the values recorded for BG/WSN_2h and BG/WSN_24h are 10.5% and 18.5%, respectively. Thus, YI for the supported biocatalysts reaches 23% in 2 h and 80% in 24 h, corresponding to 38 and 133 mg/g of support, respectively. The presented results confirm that the dilution of both enzyme and support as well as the choice for a lower BG:WSN w/w resulted in the optimization of the immobilization route. In fact, the achieved enzyme loading in 24 h was comparable to that of the reference system namely the biocatalyst similarly produced by Califano et al.^26^ using a BG:WSNs w/w ratio of 1:2 (133 mg/g vs 150 mg/g) whereas YI was more than doubled, rising from 30% to 80%. The feasibility of using TGA analysis for protein content determination was previously assessed. The BG:WSN system was tested for protein content with both TGA^26^ and the Bradford method,^31^ giving exactly the same result of 150 mg/g.

Such enhancement in YI was not unexpected. Indeed, it was observed that absorption of cellulolytic enzymes into WSNs follows a Langmuir mechanism^27^ which prescribes enzyme monolayer adsorption. According to such a mechanism, the amount of immobilized protein rises with the concentration of enzyme in solution until a plateau is reached, when all the binding sites of the support are saturated. Therefore, low enzyme concentrations lead to high YI values because YI follows an exponential decay function.

### 3.3 Conformational Analysis of Immobilized BG

**Figure 8:**
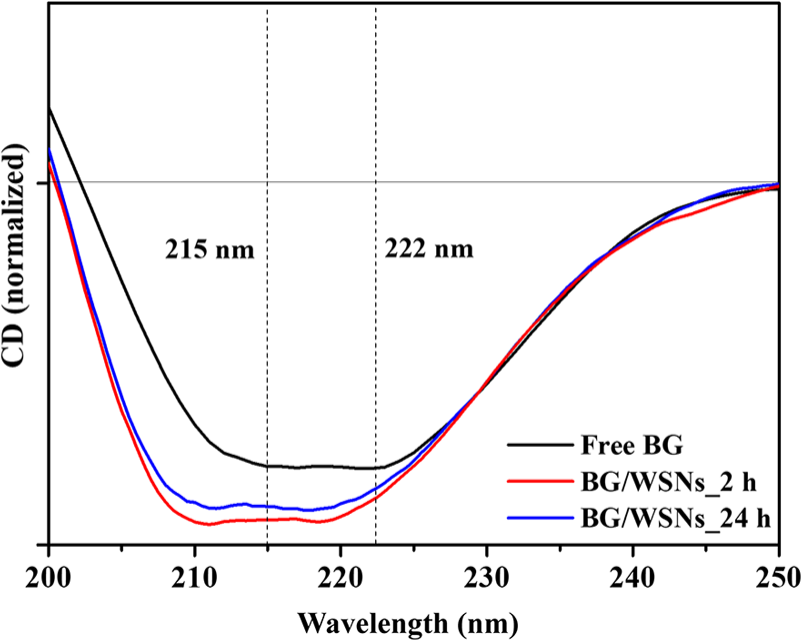
Comparison between CD spectra of free BG (black curve) and BG immobilized on WSNs after 2 h (red curve) and 24 h (blue curve).

To analyze the effect of BG immobilization on WSNs at different times on the enzyme conformation, CD spectra of free BG and BG/WSNs systems after adsorption at 2h (BG/WSN_2h) and 24h (BG/WSN_24h) were recorded, as shown in Figure 8.

**Figure 9:**
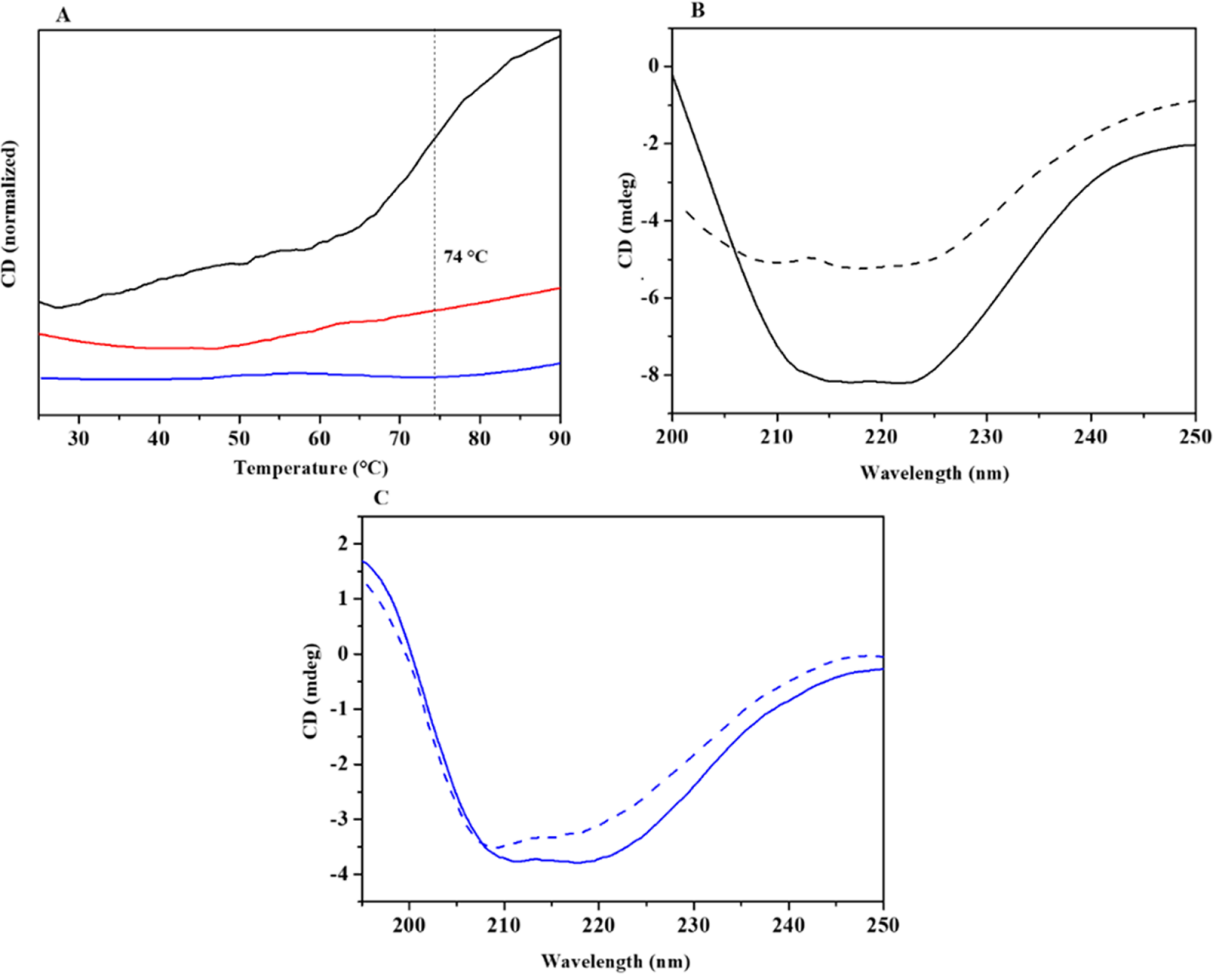
Thermal denaturation curves for free BG (black line), BG/WSNs_2h (red line), and BG/WSNs_24h (blue line) (A). Comparison between CD spectra of free BG (B) and BG/WSNs_24h (C) acquired before (dashed curve) and after (solid curve) a thermal denaturation ramp.

The spectrum of the free enzyme showed two minima centered at 215 and 222 nm, suggesting the presence of comparable amounts of β-sheet and α-helix components.^50−53^ The spectra of the BG/WSNs systems are similar but slightly different from that of the free protein. Indeed, the two minima are better resolved and fall at about 210 and 220 nm. These spectral features may suggest a slightly higher presence of α-helices with respect to β-sheets. However, the comparison between the spectra highlights that the enzyme does not unfold and retains its secondary structure when adsorbed on the nanosilica skeleton in 2 h as well as 24 h. The enzyme in its free form experienced a two-step denaturation phenomenon. In detail, the first step is likely due to rearrangements of the quaternary structure, whereas the second one to the loss of secondary structure, with a melting temperature of 74 °C. In fact, it was found that β-glucosidase from almonds exist in two isoforms, monomeric and dimeric, with the dimeric form that performs much better than the monomeric one.^54^ Thermal denaturation curves of the immobilized samples (Figure 9A) did not exhibit remarkable signs of denaturation up to 90 °C. More specifically, the thermal curve of BG/WSN_24h remains flat, indicating that no structural change occurs. Differently, the slight slope exhibited by the BG/WSN_2h thermal profile reveals a partial structural modification. Such distinct thermal behaviors could be attributed to the different protein organizations and densities onto the silica skeleton. Indeed, the protein is mostly externally adsorbed over the surface of the nanostructure after 2 h of immobilization and thus free to undergo modifications of quaternary and tertiary structure. On the contrary, the enzyme is best shielded when hosted inside the pores as for BG/WSNs_24h because the pore wall–protein physical interaction ensures conformation rigidity, resulting in more improvement of the thermal stability than BG/WSNs_2h.^31^ Thermal stabilization of enzymes is particularly important for multimeric enzymes (dimeric in our case) where dissociation of the subunits can produce inactivation.^55^ It was argued that for β-glucosidase, inactivation may start by subunit dissociation.^56^ In our case, immobilization seems to stabilize the quaternary structure of the enzyme. Stabilization of multimeric enzymes by physical adsorption was observed where multipoint enzyme–support interactions exist,^55^ due to the presence of several interacting groups on the support surface [i.e, OH for hydrogen bonding and O^–^ for electrostatic interactions).

The anchoring into the pores of WSNs dramatically improved the thermal stability of the enzyme. The benefits brought by the physical immobilization to the thermal resistance of the enzyme clearly emerge from the comparison between CD spectra of free BG and the most stable supported biocatalyst namely BG/WSNs_24h acquired before and after subjecting the sample to a denaturation test (Figure 9B, 9C). The free protein experienced a remarkable change in the 200–225 nm range and a very strong decrease in CD intensity, thus confirming that it is mostly unfolded.^57^ Differently, immobilized BG exhibited only slight variations in the spectrum profile, confirming the enhanced rigidity of the protein chains provided by the physical immobilization.

The deconvolution of the amide I band carried out by FTIR spectroscopy of BG/WSNs_24h (Figure S4) confirmed that the enzyme underwent only a little structural modification upon adsorption onto WSNs. As reported in Table 1, the obtained structural pattern underlines that the optimized system showed more similarities with the original structure of the BG with respect to that observed for the immobilized enzyme at the highest WSNs/BG weight ratio, as previously prepared acting as reference system.^26^

**Table 1:** Conformational Analysis for BG/WSNs_24h Compared to Free BG^58^ and to the Reference System^26^

structure	free form	BG/WSNs_24h	reference system (BG:WSNs = 1:2,*t* = 24 h)
aggregates		17.1	9.4
β-sheets	30	25.1	34.2
α-helices	34	30.8	20.4
turns	25	22.0	25.2
disordered	11	4.9	10.8

Indeed, the percentage of α-helices (30.8%) was higher and closer to the one exhibited by the BG in its free form (34%), just like the difference between the percentage amounts of α-helices and β-sheet,^58^ confirming that observed through CD measurements. Moreover, the non-negligible value for aggregate portions could be a consequence of protein rearrangement when adsorbed onto the nanostructure or might occur during the drying process necessary to analyze the samples by FTIR.

### 3.4 Catalytic Assays

BG/WSNs_2h and BG/WSNs_24h were both assayed in the hydrolysis of cellobiose to glucose using the same amount of immobilized enzyme. Table 2 shows the immobilization parameters and activity for the supported biocatalysts, compared to the reference system and to soluble BG.

**Table 2:** Summary of the Immobilization Parameters for the Supported Biocatalysts, Compared to the Reference System^26^ and to Soluble BG

biocatalyst	YI (%)	YI_E_ (%)^a^	load (mg/g_support_)	SA^b^(U/mg_BG_)	RA^c^(U/mg_support_)
BG/WSNs_2h	23	38.2	38	7.77	0.07
BG/WSNs_24h	80	140	133	8.22	0.28
reference system	30	54.2	150	8.44	0.32
free BG				4.67	

a The yield of immobilization expressed in terms of activities (YI_E_) was measured as the percentage ratio between the activity of the immobilized protein and the activity of the offered protein in the immobilization step.

b Specific activity (SA) is defined as the recorded activity per mass of BG.

c Recovered activity (RA) is defined as the recorded activity per mass of support.

As can be seen, all the immobilized biocatalysts show hyperactivation, possibly due to an increased concentration of the substrate near the active site.^26^ However, for the reference biocatalyst, it has been shown that the situation levels off over a longer period: there is a decrease in the rate of the reaction after 60 min for BG_WSN with respect to free BG, probably due to the accumulation of glucose inside the matrix.^26^

**Figure 10:**
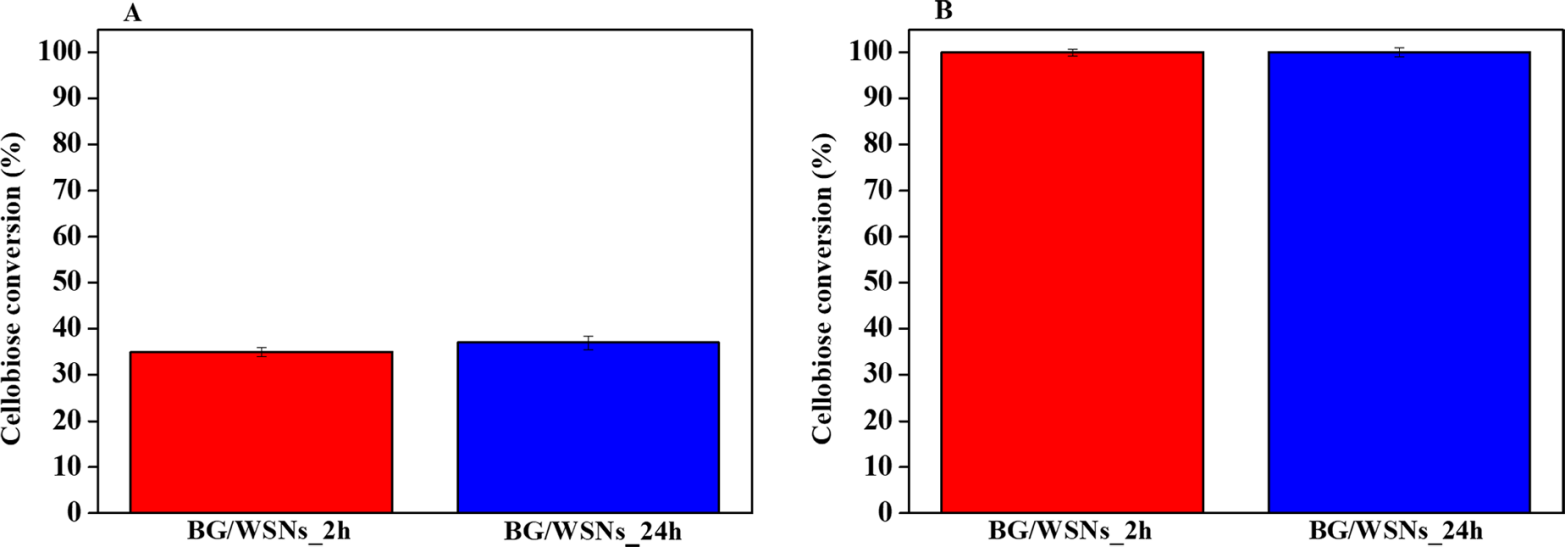
Histograms reporting cellobiose conversion (%) reached by using BG/WSNs_24h and BG/WSNs_2h for 10 min (A) and 24 h (B).

Figure 10 shows the histogram reporting the cellobiose conversion achieved by the two biocatalysts in 10 min and 24 h of reaction. Both biocatalysts allowed for about 35% cellobiose conversion after 10 min (Figure 10A). The specific activities were 7.77 and 8.22 U/mg_BG_ for BG/WSNs_2h and BG/WSNs_24h, respectively (calculated by dividing the activity values for the weight of the actual BG contained in the commercial product). Moreover, both systems pushed cellobiose conversion up to 100% in 24 h (Figure 10B). Catalytic assays thus highlight that these biocatalysts exert performance similar to that of the biocatalyst chosen as the reference (activity ∼8.44 U/mg_BG_, 100% cellobiose conversion in 24 h), produced by adsorption of BG into WSNs for 24h, fixing enzyme and support concentrations to 1 and 2 mg/mL, respectively.^26^ The obtained results confirm that assessed by CD analysis which is that the enzyme conformation is unaffected or even improved by physical immobilization, leading to performing biocatalysts produced after both 2 and 24 h of adsorption. Such achievements mean that this modified adsorption route leads to biocatalysts which retain conformation and improved activity, although using only a third of the enzyme needed previously in the immobilization step with respect to the reference system designed by Califano et al.^26^

In the end, 24 h is confirmed as the optimal immobilization time. Indeed, it allows for the highest YI (80%), resulting in a consistent enzyme saving. Moreover, enzyme location within the pores is responsible for the largest improvement in protein thermal stability, as assessed by CD analysis (Figure 9A). The 2 h adsorption leads to a transient state that is not in equilibrium. Actually, it is shown that after 24 h the protein corona disappears and the enzyme is mainly located inside the pore. Furthermore, it was found that in porous nanoparticles the proteins of the corona can undergo, during storage, intraparticle migration inside the pores.^25^ Therefore, the catalyst is likely to change over time in an uncontrollable way.

### 3.5 Operational and Thermal Stability

**Figure 11:**
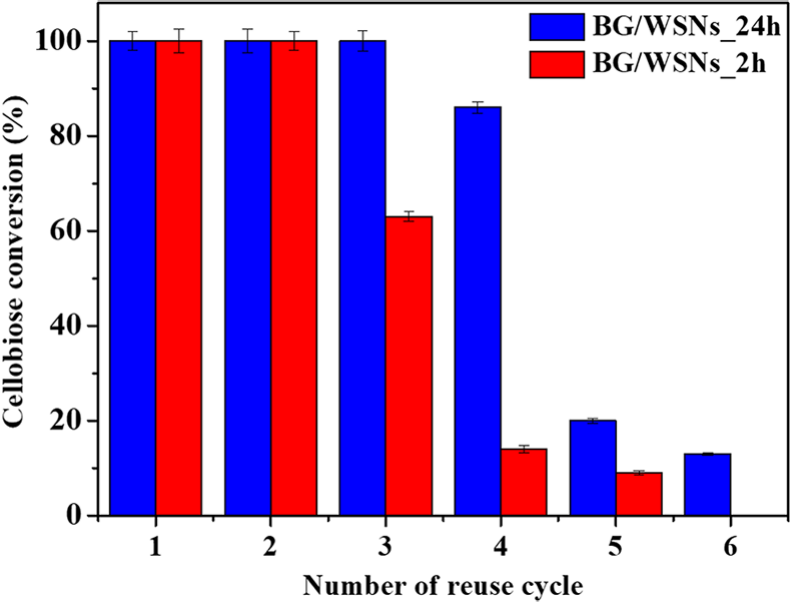
Cellobiose conversion over the number of reuse cycles for BG/WSNs_24h (blue) and BG/WSNs_2h (red).

The arrangement and organization of the protein over the porous architecture of the silica nanoparticles in the different biocatalysts affect the operational stability, due to conformational variations or leakage phenomena. As a matter of fact, BG/WSNs_24h and BG/WSNs_2h systems exert different performances in terms of reusability, as shown in Figure 11.

BG/WSNs_24h biocatalyst exhibits total reusability up to the third cycle, only losing 20% of conversion at the fourth one. Afterward, the performances of the biocatalyst drop to about 20% and 15% conversion at the fifth and sixth cycles, respectively. The operational stability of this system was already tested for the reference system: there was no loss of activity after three repeated uses. In the fourth, the yield reduced to 80% and 40% with the fifth reuse.^26^

A comparable trend is reported for BG/WSNs_2h. However, it keeps complete conversion only for two cycles, losing 40% conversion at the third one. In a way similar to that of BG/WSNs_2h, after the third cycle it experiences a fall in conversion until losing it all at the sixth reuse cycle. BG/WSNs_24h’s higher operational stability can be attributed to the penetration of BG into the pores of the nanostructure. This maximizes the protein–matrix interaction, reducing the risk of both conformational modifications and leakage phenomena. On the contrary, BG is set mostly over the outer surface of BG/WSNs_2h, being exposed to the release of the external protein layers as long as the reusability tests go on.^59^ Indeed, TGA measurements proved that the BG/WSNs_2h sample loses almost the 88% of the original enzyme load after four reaction cycles whereas only the 20% of protein is released from the pore structure of BG/WSNs_24h.

**Figure 12:**
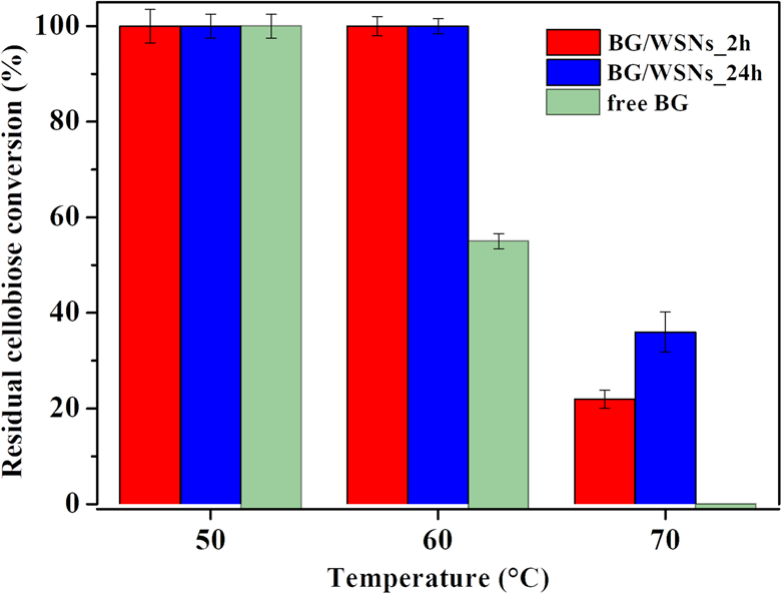
Residual cellobiose conversion (%) over the course of the incubation. Data for reaction without previous incubation (bars at 50 °C) were reported for clearer comparison.

Figure 12 shows the results of the thermal stability experiments. The bar plot highlights that the immobilized enzyme recovers higher cellobiose hydrolytic activity than that of its free counterpart upon an incubation of temperature >60 °C, regardless of the immobilization time. More specifically, free BG is completely inactivated after incubation at 70 °C. On the other hand, the enzyme immobilized for longer times (24 h) is slightly more stable than the enzyme immobilized for shorter times (2 h) when both are incubated at 70 °C. This result confirms the lower stability of the soluble enzyme compared to the immobilized one. Both supported biocatalysts experienced complete inactivation after incubation at 80 °C.

## 4 Conclusions

This work is focused on the study of the self-aggregation processes associated with the physical immobilization of BG into WSN with the aim to better control the protein–support interactions and their evolution as a function of time and enzyme concentration. Indeed, this behavior has been poorly studied, and many aspects related to the enzyme immobilization appear unclear.

In this work, the optimal adsorption conditions in terms of colloidal stability and yield of immobilization (YI) were found. Specifically, a BG:WSNs ratio equal to 1:6 wt/wt leads to the highest controllability of the system, as indicated by DLS analysis. In these conditions, the formation of a protein corona is observed at 2 h and a 23% YI resulted, as demonstrated by TEM and TGA analyses, respectively. However, the enzyme corona disappears after 24 h as the protein diffuses inward to reach the inner edge of the pores, achieving 80% YI. At the same time, the enzyme conformation was only slightly affected by physical immobilization, as confirmed by FTIR and CD measurements. Indeed, a huge gain in thermal stability of the supported enzyme was observed after both 2 and 24 h of immobilization. More specifically, BG/WSNs_24h preserves almost complete folding even at 90 °C owing to the interactions between the pore walls and the protein established as the enzyme is located inside the pores. Both BG/WSNs_2h and BG/WSNs_24h show complete conversion of cellobiose to glucose after 24 h of reaction at the same enzyme concentration, proving the success of the adsorption protocol in preserving native enzyme secondary structure. The sensitively high YI reached after 24 h points out BG/WSNs_24h to be the best obtained biocatalyst, exerting comparable performances to that of previously prepared BG:WSNs having a ratio equal to 1:2 wt/wt and about 10-fold the support concentration.^26^ However, this favorable biocatalytic activity is strongly associated with the enhanced controllability achieved as the BG:WSNs wt/wt ratio is set to 1:6 and the protein amount is lowered by 1 order of magnitude, also guaranteeing a noticeable enzyme saving. Moreover, the new adsorption protocol results in a fully reusable biocatalyst up to the fourth cycle of reaction.

In summary, the proposed study underlines the key role of a fine-tuning of immobilization processes, in terms of both time and enzyme content onto inorganic supports, to improve colloidal stability and to prevent fast self-aggregation processes as decisive strategies to enhance the enzyme loading and reduce protein waste without undermining the biocatalytic performances.

## Supporting Information Available

Figure S1: Absorption spectrum for 1 mg/mL BG buffer solution. Figure S2: SDS PAGE images for offered BG (a), supernatant (b), and immobilized BG (c). Figure S3: Autocorrelation functions of BG:WSNs weight ratios of 1:2 (pink line), 1:4 (magenta line), 1:6 (violet line), and 1:10 (purple line) recorded after 24 h. Figure S4: Gaussian deconvolution of amide I peak for BG/WSNs_24 h sample (PDF)
