# Supplementary material for: Controlling the Adsorption of β-Glucosidase onto Wrinkled SiO2 Nanoparticles To Boost the Yield of Immobilization of an Efficient Biocatalyst
Source: Langmuir. 2023 Jan 18;39(4):1482–94. doi: 10.1021/acs.langmuir.2c02861 (PMC9893809; doi:10.1021/acs.langmuir.2c02861)
Supplement: Supplementary file 1 — la2c02861_si_001.pdf [file la2c02861_si_001.pdf]

Supporting information for:

Controlling the adsorption of β-glucosidase onto wrinkled SiO_2 nanoparticles to boost the yield of immobilization of an efficient biocatalyst

Giulio Pota[1], Noemi Gallucci[2,3], Domenico Cavasso[2], Irene Russo Krauss[2,3], Giuseppe Vitiello[1,3]*, Fernando López-Gallego[4,5], Aniello Costantini[1]*, Luigi Paduano[2,3], Valeria Califano[6]

[1] University of Naples Federico II, Department of Chemical, Materials and Production Engineering, Naples, Italy.

[2] University of Naples Federico II, Department of Chemical Sciences, Naples, Italy.

[3] CSGI, Center for Colloid and Surface Science, Sesto Fiorentino (FI), Italy.

[4] Center for Cooperative Research in Biomaterials (CIC BiomaGUNE), Basque Research and Technology Alliance (BRTA), Donostia-San Sebastián, Spain;

[5] Ikerbasque, Basque Foundation for Science, Bilbao, Spain;

[6] Institute of Sciences and Technologies for Sustainable Energy and Mobility (STEMS), National Research Council of Italy (CNR), Viale Marconi 4, 80125 Naples, Italy.

* Correspondence: giuseppe.vitiello@unina.it, anicosta@unina.it

S.1. Quantification of the enzyme fraction in commercial BG

The recorded spectrum exhibits a single peak centered at around 280 nm, with absorbance of 0.17 (Figure S1). The actual protein molar concentration was calculated by using equation (1) and the estimated value was 1.78×10^{-6} M, being ε and l equal to 95310 $cm^{-1}M^{-1}$ [1] and 1 cm, respectively, corresponding to a mass concentration of 0.24 mg/mL. Thus, only the 24% of the commercial product can be attributed to enzyme macromolecules.

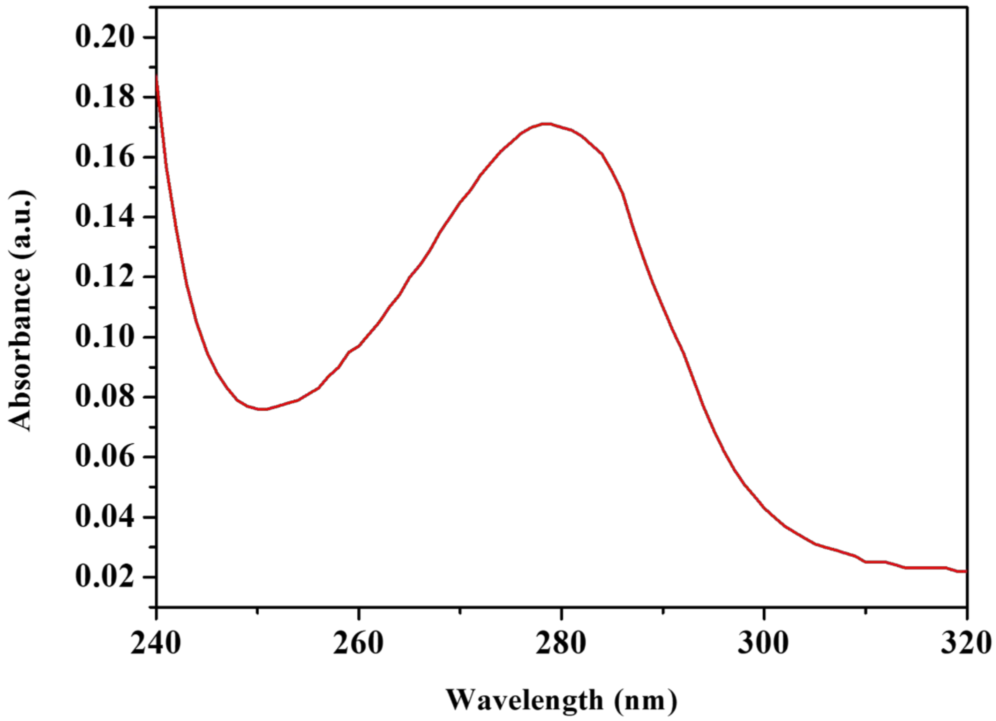

Figure S1. Absorption spectrum for 1 mg/mL BG buffer solution

S.2. Sodium Dodecyl Sulphate - PolyAcrylamide Gel Electrophoresis (SDS-PAGE)

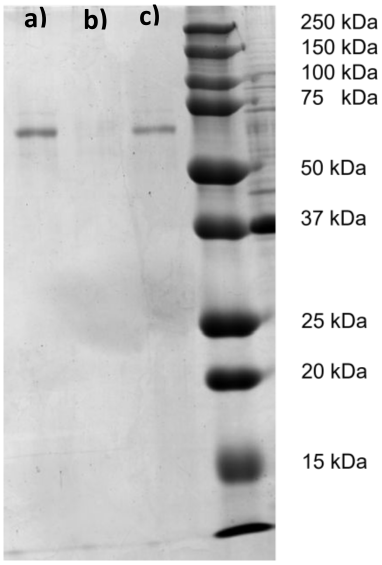

Figure S2. SDS PAGE images for offered BG (a), supernatant (b), immobilized BG (c).

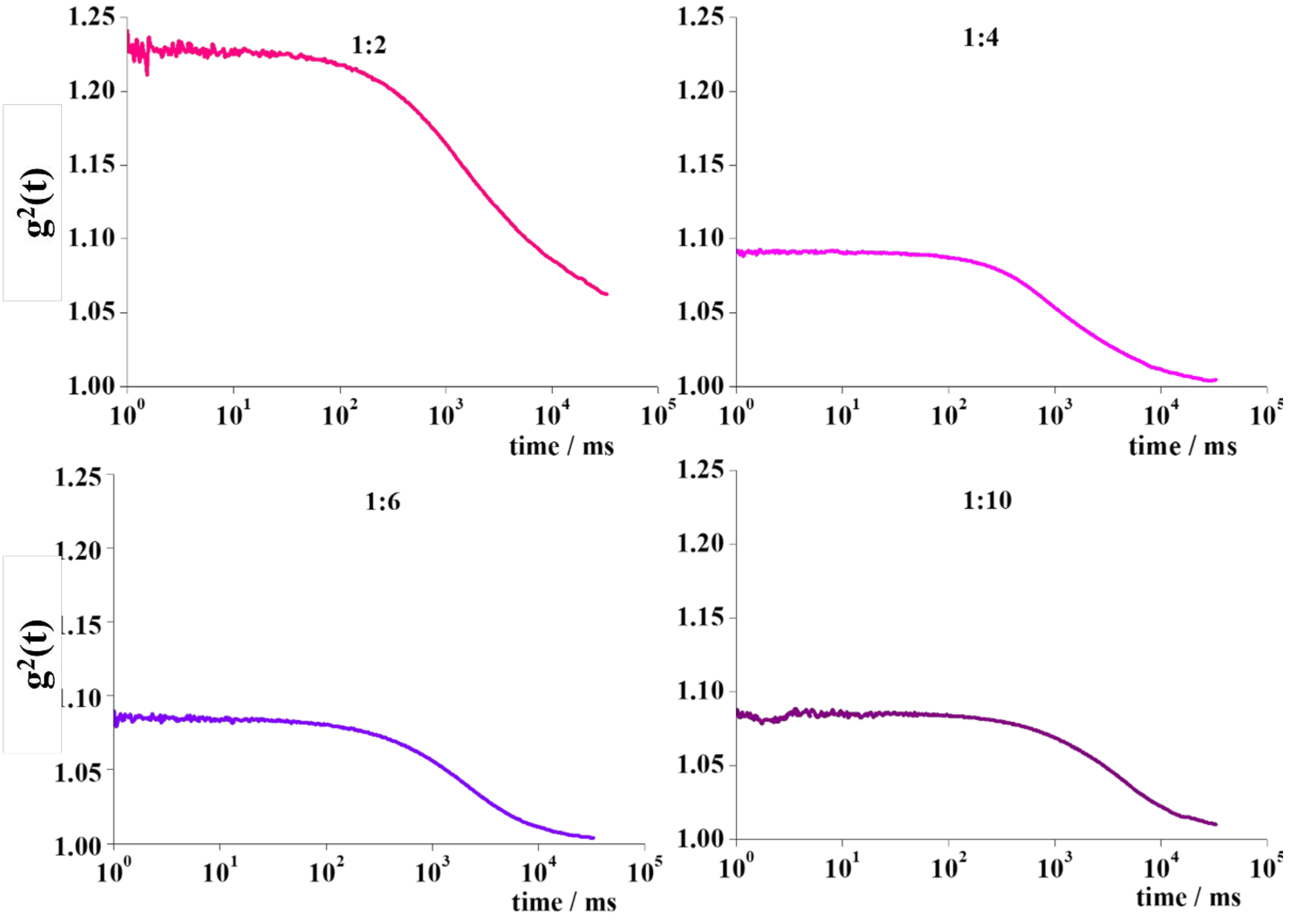

Figure S3. Autocorrelation functions of BG:WSNs weight ratios of 1:2 (pink line), 1:4 (magenta line), 1:6 (violet line), 1:10 (purple line) recorded after 24 h.

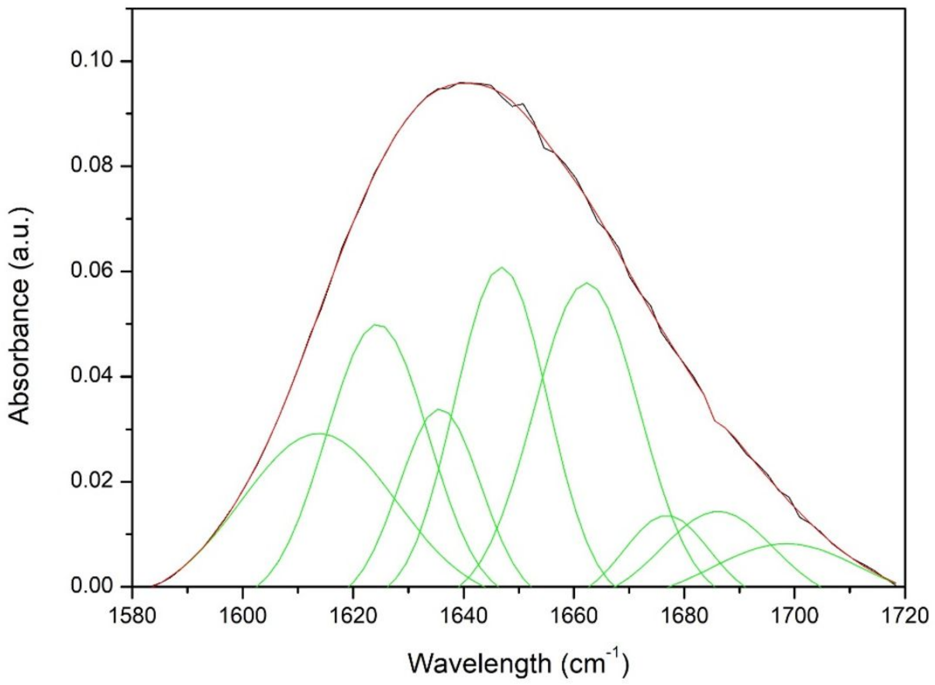

Figure S4. Gaussian deconvolution of Amide I peak for BG/WSNs_24 h sample

References

[1] A.K. Grover, D.D. MacMurchie, R.J. Cushley, Studies on almond emulsin βd-glucosidase I. Isolation and characterization of a bifunctional isozyme, Biochimica et Biophysica Acta (BBA)-Enzymology. 482 (1977) 98–108.
